# Microbial and metabolic crosstalk in the rhizosphere shapes the divergent drought resilience of contrasting rice genotypes

**DOI:** 10.3389/fmicb.2026.1788826

**Published:** 2026-04-29

**Authors:** Jinwei Qi, Kehui Zhang, Chengfang Zhan, Xueli Lu, Xujie Chen, Xinyao Li, Chengyi Zhang, Hui Wang, Chaohuang Tu, Wenjin Tong, Liping Dai, Dali Zeng

**Affiliations:** College of Advanced Agricultural Sciences, Zhejiang A&F University, Hangzhou, China

**Keywords:** drought stress, genotype-dependent responses, metabolomics, metagenomics, rhizosphere metabolites, rhizosphere microbiome, rice

## Abstract

Drought is a major constraint on rice production, yet the coordinated responses of rhizosphere microbial communities and metabolites across rice genotypes with contrasting drought tolerance remain insufficiently understood. In this study, we combined metagenomic and metabolomic analyses to investigate drought-induced changes in the rhizosphere of three rice genotypes with distinct ecological backgrounds: the drought-sensitive cultivar Bhutan, the upland rice genotype TGR78, and *Oryza rufipogon* K111. Field experiments were conducted under well-watered and drought conditions, and rhizosphere soil samples were collected for multi-omics profiling. Drought stress reduced plant height and panicle number in all three genotypes, but the magnitude of these effects differed among genotypes. Bhutan showed the greatest reduction in plant height (42.1%) and the largest number of differential metabolites (146), indicating a stronger drought response at both phenotypic and metabolic levels. In contrast, TGR78 and K111 displayed relatively greater phenotypic stability under drought stress. Metagenomic analysis revealed pronounced genotype-dependent shifts in rhizosphere bacterial community composition, whereas metabolomic profiling showed distinct changes in metabolite accumulation patterns among genotypes. Correlation analysis further demonstrated that drought substantially reshaped rhizosphere microbe–metabolite associations, shifting the interaction network from broadly positive and highly connected under well-watered conditions to more selective associations under drought stress. Collectively, these results indicate that rice drought adaptation is associated with genotype-dependent reorganization of the rhizosphere microbiome and metabolic profile. This study provides new insight into rhizosphere-mediated drought responses in rice and offers a basis for developing microbiome-informed strategies for drought-resilient crop improvement.

## 1 Introduction

Drought is one of the most severe abiotic stresses limiting agricultural productivity worldwide and poses a major threat to food security, particularly for rice (*Oryza sativa* L.), which feeds more than half of the global population. As a water-intensive crop with flood-adapted physiology, rice is especially vulnerable to water deficit (Lesk et al., 2016). Under ongoing climate change, the frequency and intensity of drought events are expected to increase, further exacerbating yield instability in rice-growing regions (Trenberth et al., 2014). Drought stress disrupts a range of physiological and biochemical processes in rice, including water and nutrient uptake, carbon assimilation, hormone homeostasis, and overall growth, ultimately leading to reductions in biomass accumulation and grain yield (Barnabás et al., 2008; Farooq et al., 2009; Comas et al., 2013).

Rice genotypes vary substantially in their responses to drought stress, and this natural variation provides an important foundation for the development of drought-resilient cultivars. Drought-tolerant genotypes can adopt multiple adaptive strategies, such as osmotic adjustment, modification of root system architecture, and activation of stress-responsive genes, thereby alleviating the negative effects of water limitation on plant growth and productivity (Vikram et al., 2011). Considerable progress has been made in identifying loci, candidate genes, and physiological traits associated with drought adaptation in rice, providing valuable resources for marker-assisted selection and molecular breeding (Bernier et al., 2008; Kumar et al., 2008; Venuprasad et al., 2009; Henry et al., 2011; Todaka et al., 2017). Beyond plant-intrinsic mechanisms, emerging evidence highlights the role of the rhizosphere microbiome in mediating drought responses. The rhizosphere harbors diverse microbial communities that influence nutrient cycling, pathogen suppression, and stress tolerance (Berendsen et al., 2012; Lundberg et al., 2012; Peiffer et al., 2013). Under drought conditions, the composition and function of these communities often shift, with drought-tolerant taxa potentially enhancing plant resilience through mechanisms such as phytohormone production, osmolyte accumulation, and improved nutrient mobilization (Marulanda et al., 2009; Rolli et al., 2015; Vurukonda et al., 2016; Naylor and Coleman-Derr, 2018;). These findings suggest that the rhizosphere microbiome is an integral component of plant drought resistance (Yang et al., 2009; Fadiji et al., 2023).

A key determinant of rhizosphere microbial assembly is the composition of root exudates-a complex mixture of sugars, amino acids, organic acids, and secondary metabolites that serve as nutrients and signaling molecules for soil microorganisms (Bais et al., 2006; Badri and Vivanco, 2009). Drought stress alters exudation patterns, which in turn can reshape microbial community structure and function (Chaparro et al., 2014; Shi et al., 2016; Williams and de Vries, 2020). Moreover, genotype-specific differences in exudate profiles may drive the recruitment of distinct microbial taxa, potentially contributing to differential drought adaptation among rice varieties (Micallef et al., 2009; Zhalnina et al., 2018; Bakker et al., 2020). Although plant genotype, rhizosphere microorganisms, and root-associated metabolites have each been implicated in drought responses, their coordinated relationships remain insufficiently understood, particularly in rice. It is unclear how contrasting drought-tolerant genotypes differ in their rhizosphere microbial assemblages and metabolite profiles under drought stress, and how these two components are linked in mediating stress adaptation (Sasse et al., 2018; Toju et al., 2018; Jansson and Hofmockel, 2020; de Vries et al., 2020). Addressing this gap is essential for elucidating the ecological basis of drought resilience and for identifying rhizosphere-associated traits that may be exploited in sustainable crop improvement.

In the present study, we used metagenomic and metabolomic approaches to investigate drought-induced changes in the rhizosphere of three rice genotypes with contrasting drought tolerance. Specifically, we aimed to: (1) characterize the diversity and composition of rhizosphere microbial communities under control and drought conditions using metagenomic sequencing; (2) profile drought-responsive rhizosphere metabolites using ultra-performance liquid chromatography–tandem mass spectrometry (UPLC-MS/MS) (Want et al., 2013); and (3) examine the relationships between microbial community shifts and metabolite changes across genotypes. By integrating microbial and metabolic data, this study provides new insight into genotype-dependent rhizosphere responses to drought stress and offers a basis for exploring plant-microbiome interactions in rice drought-resistance improvement (Pascale et al., 2020).

## 2 Materials and methods

### 2.1 Experimental conditions and design

The field experiment was conducted from April to May 2024 in Lingshui County, Hainan Province, China (18°30′N, 110°02′E), to evaluate the drought responses of three rice genotypes: common wild rice (K111), upland rice (TGR78), and conventional cultivated rice (Bhutan). The experimental site is located in a tropical monsoon climate zone, characterized by warm temperatures, high humidity, and seasonal rainfall. In April 2024, the average temperature in Lingshui was approximately 29 °C, with average daily maximum and minimum temperatures of about 32 °C and 26 °C, respectively. The average precipitation in May was approximately 98.7 mm, and the relative humidity was around 80%. The soils are generally acidic to weakly acidic.

The experiment was arranged in a randomized complete block design with two water regimes (control: 80–90% field capacity; drought: 35–45% field capacity) and three biological replicates. All experimental plots received consistent agronomic management practices (including uniform fertilization and pest control) to ensure that observed differences were primarily attributable to water stress.

### 2.2 Plant agronomic trait determination

To evaluate the growth responses of different rice genotypes under drought stress, plant height and tiller number were measured at the heading stage. Fifteen representative plants were randomly selected from the middle row of each plot as replicates (*n* = 15). Plant height was measured with a tape measure, from the base of the plant to the top of the highest panicle (excluding the awn), with precision to 0.1 cm. Tiller number was determined by manually counting the number of effective tillers per plant. All data were recorded, and means and standard errors were calculated for subsequent statistical analysis.

### 2.3 Rhizosphere soil sample collection

Rhizosphere soil samples were collected at the heading stage when drought stress reached the threshold level. Sampling was conducted between 9:00 and 11:00 a.m. to minimize diurnal variation. Several healthy plants were randomly selected from the central rows of each plot, avoiding side rows and diseased plants. Root balls surrounding the root systems (approximately 15–20 cm in diameter and 0–20 cm in depth) were carefully excavated and placed in sterile disposable trays. Loose soil was first discarded as non-rhizosphere soil and kept separately.

Soil tightly adhering to the root surface (<1–2 mm) was collected using powder-free sterile gloves, aided by a sterile soft-bristled brush or disposable spatula. Approximately 2–5 g of soil was collected per plant. Samples from the same plot were combined and homogenized to constitute one biological replicate, yielding a total of 10–20 g of soil. After each replicate operation, all apparatus were wiped with 75% alcohol and rinsed with distilled water. Samples were collected in sterile centrifuge tubes, immediately snap-frozen in liquid nitrogen, and placed in an insulated box with dry ice for rapid on-site processing (within 5 min, according to field protocols for soil microbiomes). Finally, samples were stored at −80 °C to avoid repeated freeze–thaw cycles.

### 2.4 Metagenomic sequencing and analysis

#### 2.4.1 DNA extraction, library construction, and sequencing

Total genomic DNA was extracted from 0.5 g soil samples using the TGuide S96 magnetic bead-based soil/fecal DNA extraction kit (Biomarker Technologies, Beijing, China) according to the manufacturer’s protocol. DNA concentration and purity were assessed using a NanoDrop 2000 spectrophotometer (Thermo Fisher Scientific, Wilmington, DE, USA) and a Qubit 3.0 fluorometer (Invitrogen, Carlsbad, CA, USA), while integrity was checked by 1% agarose gel electrophoresis. Only DNA samples with an OD_260nm/280nm_ ratio between 1.8–2.2, an OD_260nm/230nm_ ratio between 1.8–2.5, and an intact band (>23 kb) were used for library construction.

Sequencing libraries were prepared using the VAHTS Universal Plus DNA Library Prep Kit for Illumina (Vazyme, Nanjing, China) following the standard protocol. Briefly, 10 ng of DNA was fragmented, end-repaired, A-tailed, and ligated to Illumina adapters. After magnetic bead-based purification and size selection, PCR amplification was carried out for 8 cycles. The final libraries were quality-checked using a Qsep-400 for fragment distribution (expected peak 430–530 bp) and quantified by Qubit 3.0. Qualified libraries were sequenced on the Illumina NovaSeq 6,000 platform (Illumina, San Diego, CA, USA) using a paired-end 150 bp (PE150) strategy.

### 2.5 Metabolome determination and analysis

#### 2.5.1 Metabolites extraction

The LC/MS system for metabolomics analysis consisted of an Ultim3000 high-performance liquid chromatograph coupled with an Orbitrap Exploris 480 high-resolution mass spectrometer. The chromatographic column used was a Waters Acquity UPLC HSS T3 column (1.8 μm, 2.1 × 100 mm). For positive ion mode: mobile phase A was 0.1% formic acid in water, mobile phase B was 0.1% formic acid in acetonitrile. For negative ion mode: the mobile phase composition was the same as in positive ion mode. The injection volume was 1 μL.

#### 2.5.2 LC–MS/MS analysis

The Q Orbitrap mass spectrometer was used for its ability to acquire MS/MS spectra on a data-dependent basis (DDA) during an LC/MS experiment. The selected scanning range 67–1,000. The parameters of the ESI ion source were as follows: Spray voltage: 3500 V (positive ion mode) or –2500 V (negative ion mode); Sheath gas flow rate: 50 arb; Aux Gas flow rate: 10 arb; Sweep gas flow rate: 1arb; Ion Transfer Tube Temp: 325 °C; Vaporizer Temp 350 °C.

#### 2.5.3 Metabolome data preprocessing and annotation

Raw data (.raw) acquired by LC–MS were imported into CD search library software (CD search Library, Colorado, USA) for peak extraction, peak alignment, and retention time correction. Peak alignment was based on a retention time deviation of 0.2 min and a mass deviation of 5 ppm; peak extraction was based on a mass deviation of 5 ppm, a signal intensity deviation of 30%, S/N ≥ 3, signal intensity ≥ 100,000, and adduct ion information. Metabolite identification data were obtained by searching the mzCloud, MZvault, and Chemspider databases.

### 2.6 Data integration and statistical analysis

#### 2.6.1 Metagenomic data analysis

Raw sequencing data were subjected to quality control and filtering to obtain clean data for subsequent analyses. Based on the taxonomic annotation results, the structure and composition of the microbial communities were systematically characterized. During the analysis, taxonomic profiling focused on the dominant bacterial communities within the samples, thereby reducing the potential confounding effects of other microbial taxa. Principal Component Analysis (PCA) and Non-metric Multidimensional Scaling (NMDS) were employed to evaluate the overall distribution patterns of microbial communities across different sample groups. Unweighted Pair-group Method with Arithmetic means (UPGMA) hierarchical clustering was performed to reveal the similarity relationships of community structures among samples. Alpha diversity indices, including Shannon, Simpson, and Chao1, were calculated to assess species richness and evenness within each sample. A microbial co-occurrence network was constructed to explore the association patterns among key genera, while Venn diagrams were utilized to visualize the shared and unique microbial taxa under different genotype and treatment conditions. Finally, Linear discriminant analysis Effect Size (LEfSe) was applied to identify biomarkers with significant abundance differences, using a threshold of |LDA score| > 2 and *p* < 0.05.

#### 2.6.2 Metabolomic data analysis

Analyses were performed after normalizing the original peak areas by total peak area. Principal Component Analysis (PCA) and Spearman correlation analysis were used to assess the repeatability within sample groups. Identified metabolites were searched against the KEGG, HMDB, and LipidMaps databases to obtain classification and pathway information. Fold changes were calculated based on grouping information, and the significance (*p*-value) of differences for each substance was determined using a T-test. Screening criteria were fold change > 1, *p* < 0.01, and VIP > 1. An OPLS-DA model was constructed using the R package ropls, and its reliability was validated by a 200-time permutation test. VIP values were calculated through multiple cross-validations. Differential metabolites were screened by combining fold change, *p*-value, and VIP value from the OPLS-DA model. The significance of KEGG pathway enrichment for differential metabolites was assessed using a hypergeometric distribution test.

#### 2.6.3 Microbiome-metabolomic association analysis

To explore the associations between microbial communities and metabolites, we constructed a correlation matrix based on Spearman’s rank correlation coefficient. Metagenomic data were used to extract the genus-level relative abundances of microbial genera, and metabolomic data provided the corresponding metabolite peak areas. Samples were divided into control (CK) and drought (DR) groups, and genus-level abundances were matched with metabolite peak areas to create a microbial abundance matrix and a metabolite abundance matrix.

Spearman’s rank correlation coefficient was calculated for each treatment group (CK and DR) to assess monotonic relationships between microbial genera and metabolites. Significant correlations (|r| > 0.6, *p* < 0.05) were retained to construct a microbe-metabolite interaction network.

A heatmap was generated using the pheatmap package in R (version 4.2.1), with hierarchical clustering applied to microbial genera and metabolites based on Euclidean distance and Ward. D2 linkage. The color gradient ranged from blue (negative correlation) to red (positive correlation). An annotation bar was added to distinguish between control (green) and drought (orange) treatments.

#### 2.6.4 Statistical analysis

Statistical analysis of the data presented in the figures was performed using One-way ANOVA followed by Tukey’s test for post-hoc pairwise comparisons between groups, as indicated by the different lowercase letters assigned to the bars. This was used to assess differences in plant height, panicle number, and diversity indices (e.g., Simpson index, Shannon index). For analysis of microbiome or metabolome data, Principal Component Analysis (PCA) and Venn diagram analysis were employed to explore the distribution of samples and their groupings. Further, correlation networks and bar plots were used to visualize the relationships between key factors or features. All data were analyzed using GraphPad Prism version 10 and R software (version 4.2.1) for advanced statistical analyses and visualizations.

## 3 Results

### 3.1 Effects of drought on the phenotypes of different rice genotypes

**Figure 1:**
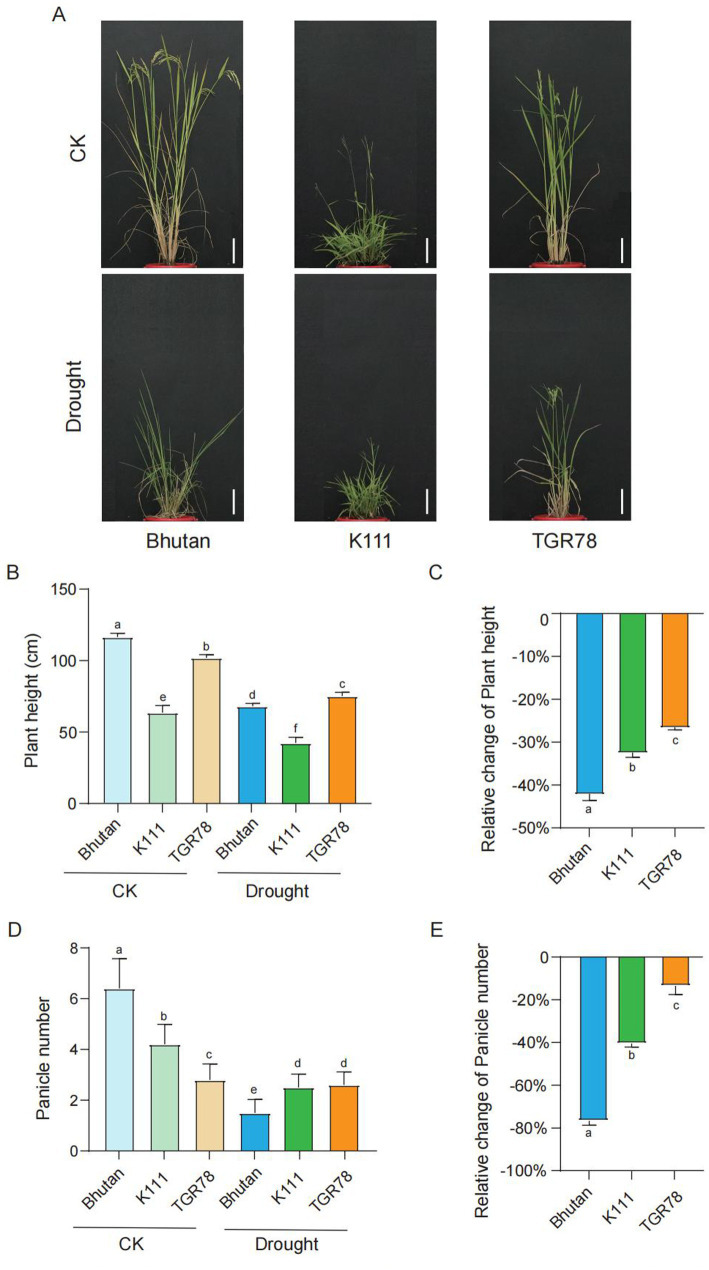
Plant phenotypic and related data analysis **(A)** Field phenotype, bars 10 cm, **(B)** Plant height, **(C)** Relative change of plant height, **(D)** Panicle numbers, **(E)** Relative change of panicle numbers, error bars represent standard deviation (*n* = 15). Bars assigned different lowercase letters are significantly different from each other (*p* < 0.05) according to Tukey’s test.

Three rice genotypes representing distinct ecological and evolutionary backgrounds were selected to evaluate their responses to drought stress. Bhutan, a traditional rice cultivar; K111, an *Oryza rufipogon* genotype, and TGR78, a typical upland rice variety. Phenotypic analysis revealed that drought stress significantly affected the growth and development of all three genotypes, albeit to different extents (Figure 1A).

Under well-watered conditions, Bhutan exhibited the greatest plant height, followed by TGR78 and K111 (Figure 1B). Drought stress reduced plant height in all three genotypes; but the magnitude of reduction varied considerably. Bhutan showed the largest decrease (42.1%), indicating pronounced growth inhibition under drought. In contrast, TGR78 displayed a comparatively smaller reduction (26.6%), while K111 exhibited an intermediate response (Figure 1C).

Panicle number also differed among genotypes under control conditions, with Bhutan producing the highest number of panicles, followed by K111 and TGR78 (Figure 1D). Following drought treatment, panicle number decreased across all genotypes. TGR78 showed the smallest reduction, Bhutan exhibited the greatest decline, and K111 again demonstrated an intermediate response (Figure 1E). Collectively, these results indicate that while Bhutan outperformed the other genotypes under well-watered conditions, it was more susceptible to drought stress. Conversely, TGR78 and K111 exhibited greater phenotypic stability under water-deficit conditions.

### 3.2 Rhizosphere microbial community structure and assembly processes

**Figure 2:**
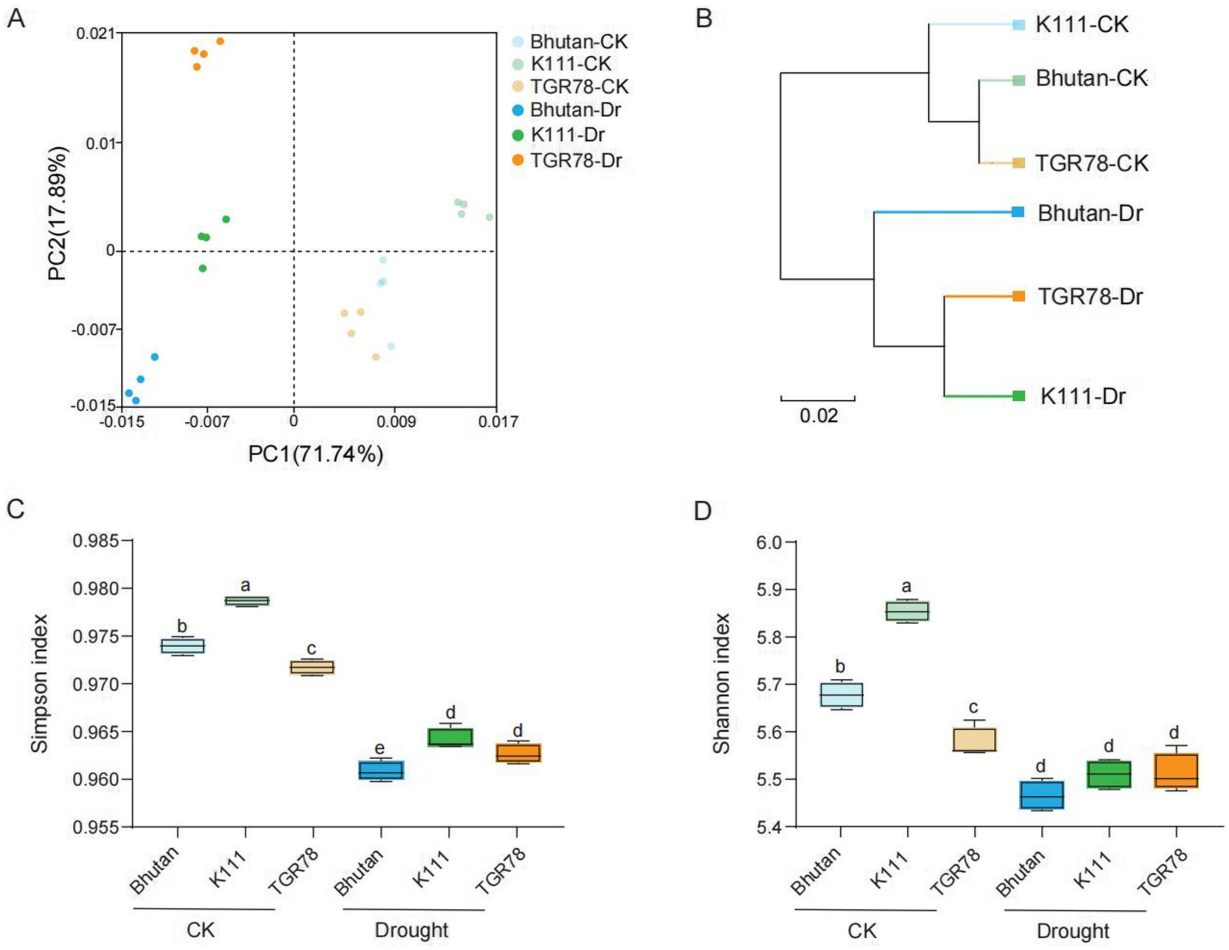
Microbial community diversity analysis. **(A)** Visualizing the distribution of microbial communities in rice rhizosphere soil using PCA. **(B)** Hierarchical clustering using the unweighted pair group method with arithmetic mean (UPGMA) revealed distinct clustering patterns among the samples, reflecting differences in their microbial community structures. **(C,D)** Boxplots show alpha diversity indices (Shannon and Simpson), indicating variation in community diversity among treatments.

**Figure 3:**
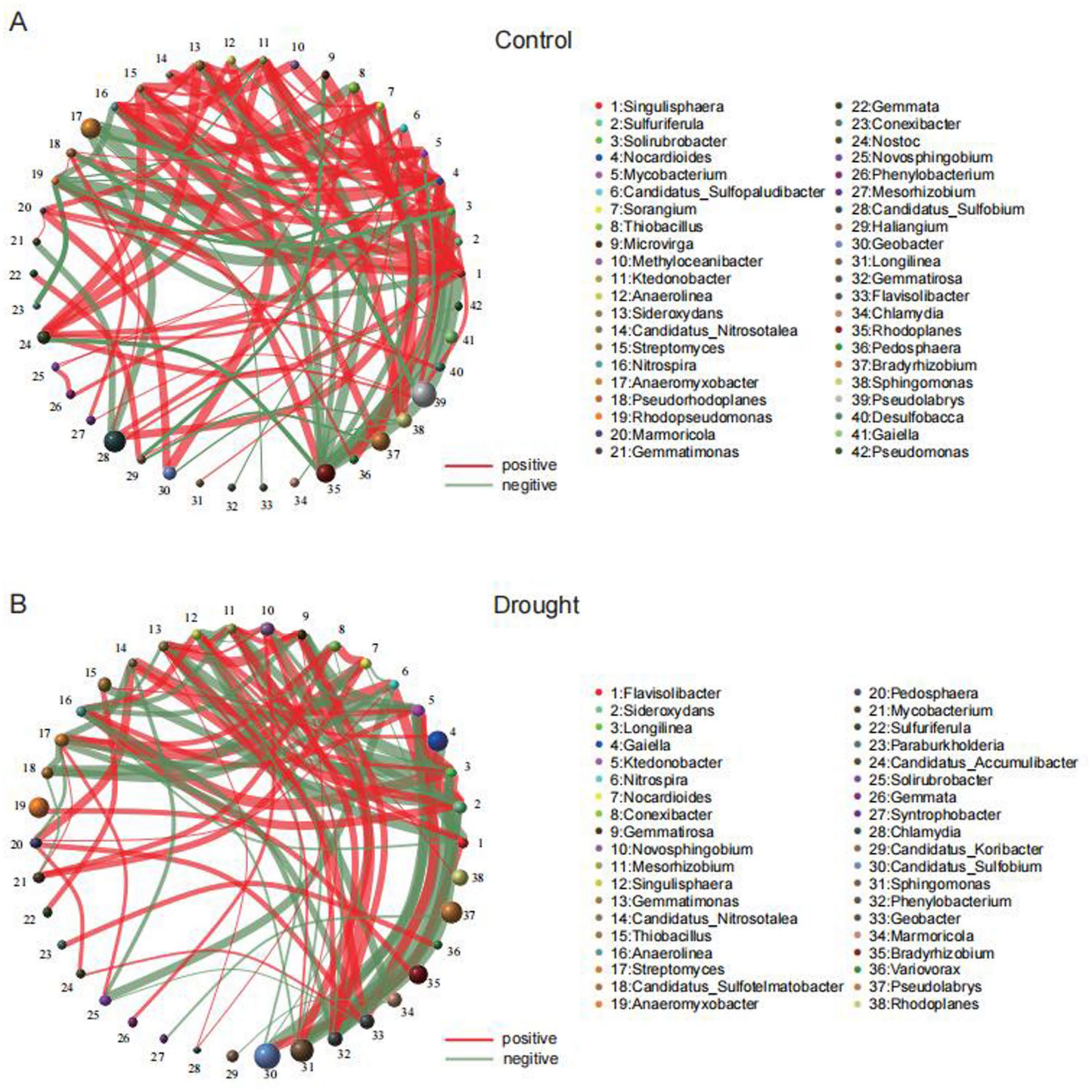
Microbial co-occurrence network in rice rhizosphere under control and drought conditions. **(A)** The network in the control condition shows microbial interactions with a relatively balanced pattern of both positive (red edges) and negative (green edges) correlations between microbial genera. **(B)** Under drought stress, the network becomes more fragmented and complex, with a noticeable increase in negative correlations, indicating disruptions in beneficial microbial interactions and the emergence of more antagonistic relationships within the microbial community. The size of each circle is proportional to the relative abundance of the microbial genus.

Metagenomic sequencing of rhizosphere samples from three rice genotypes under control and drought conditions generated 38.19–45.60 Gb of clean data per group, corresponding to 582–717 million clean reads. The K111-CK group yielded the highest data output (44.21 ± 1.54 Gb), which was significantly higher than the lowest-yielding group (39.45 ± 1.23 Gb, *p* = 0.02). No significant differences were observed among other group comparisons. Sequencing quality was consistently high across all samples, with Q30 values exceeding 93% and coefficients of variation below 5% among biological replicates (Supplementary Table S1). Functional annotation against GO and KEGG databases revealed enrichment of genes associated with carbohydrate metabolism, amino acid metabolism, energy metabolism, signal transduction, cellular processes, cellular growth, and genetic information processing (Supplementary Figures S1A,B). Principal component analysis (PCA) of microbial community composition demonstrated that PC1 and PC2 explained 89.63% of the variation attributed to drought, with control and drought-treated samples clearly separated along PC1, indicating that water availability was the primary driver of community structure (Figure 2A). Genotype-specific clustering patterns were also evident, suggesting an influence of host genetic background on rhizosphere microbial assembly. Non-metric multidimensional scaling (NMDS) analysis corroborated these findings, showing distinct clustering of control and drought-stressed samples (Supplementary Figure S2A). Unweighted pair group method with arithmetic mean (UPGMA) hierarchical clustering further revealed that all control samples (K111-CK, TGR78-CK, Bhutan-CK) formed a cohesive cluster, while drought-stressed samples segregated according to drought tolerance level. Specifically, samples from the tolerant genotypes TGR78 and K111 (TGR78-T and K111-T) clustered together, whereas samples from the sensitive genotype Bhutan (Bhutan-Dr) formed a distinct outgroup (Figure 2B). Alpha diversity indices, including Shannon, Simpson, and Chao1, revealed significant differences in rhizosphere microbial diversity among rice ecotypes following drought stress (Figures 2C,D; Supplementary Figure S2B). Microbial co-occurrence network analysis revealed differences between control and drought conditions. Under control conditions, the rhizosphere network contained both positive and negative correlations among microbial genera (Figure 3A). Under drought stress, the network became more fragmented and included a greater number of negative correlations (Figure 3B). These results indicate that drought altered the structure of microbial associations in the rhizosphere. Microbial co-occurrence network analysis revealed distinct association patterns under control and drought conditions. Under well-watered conditions, the rhizosphere network exhibited both positive and negative correlations among microbial genera (Figure 3A). Under drought stress, the network became more fragmented with an increased proportion of negative correlations (Figure 3B).

**Figure 4:**
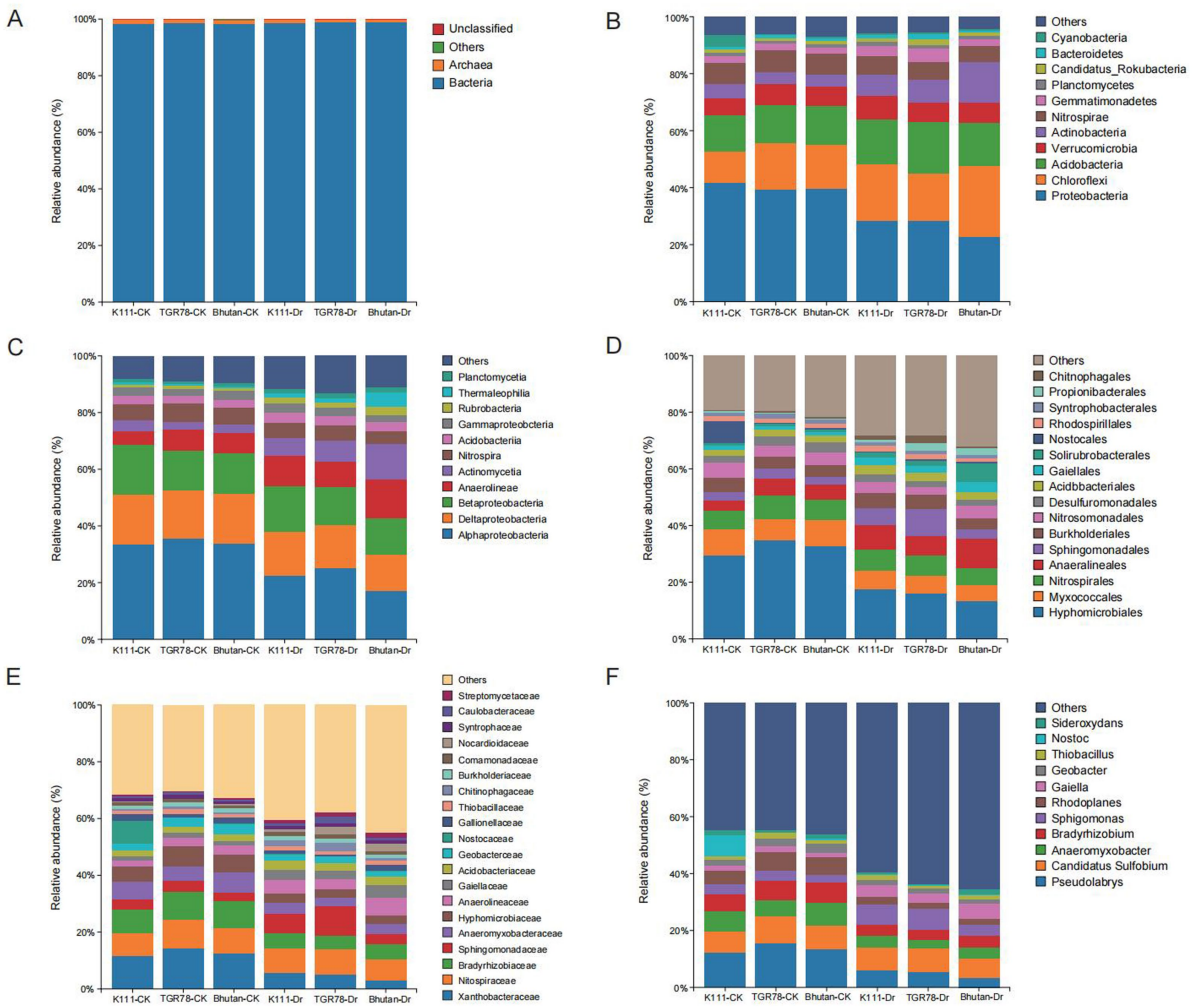
Metataxonomic analysis of rice rhizosphere microbial communities under drought stress. Relative abundance of microbial taxa (≥0.01%) at different classification levels: **(A)** Kingdom, **(B)** Phylum, **(C)** Class, **(D)** Order, **(E)** Family, and **(F)** Genus. Bar plots illustrate compositional differences across K111-CK, TGR78-CK, Bhutan-CK, K111-Dr, TGR78-Dr, and Bhutan-Dr groups.

**Figure 5:**
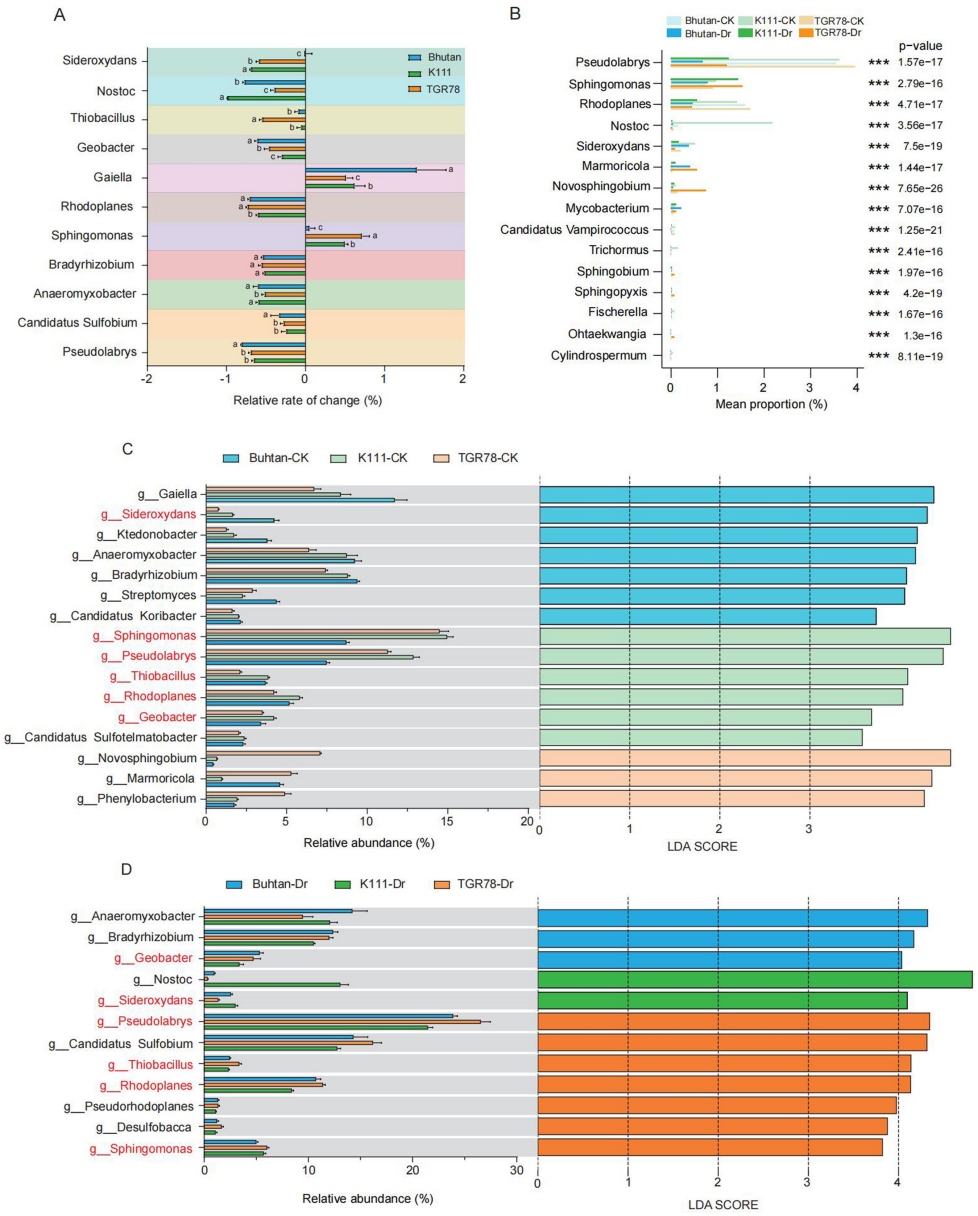
Genotype-dependent relative changes and differential abundances of rhizosphere bacterial genera under drought stress. **(A)** Relative rate of change in the abundance of dominant rhizosphere bacterial genera in three rice cultivars following drought treatment. Bars show the mean relative rate of change (%) in genus abundance under drought compared with the corresponding control, with negative values indicating a decrease and positive values indicating an increase in abundance. **(B)** Differentially abundant bacterial genera in the rhizosphere of three rice genotypes under control (CK) and drought (Dr) conditions, with significant differences indicated by *p*-values (adjusted *p* < 0.05). LDA score plots showing differential abundance of rhizosphere microbial genera across three rice genotypes under control **(C)** and drought **(D)** conditions (LDA > 3.5). Genera with significant differences are highlighted.

Metataxonomic analysis identified 9 kingdoms, 200 phyla, 177 classes, 831 families, 3,415 genera, and 21,628 species across all samples. Bacteria predominated at the kingdom level, with *Proteobacteria*, *Chloroflexi*, and *Acidobacteria* as the dominant phyla, and *Alphaproteobacteria*, *Deltaproteobacteria*, and *Betaproteobacteria* as the predominant classes (Figures 4A–F). Drought stress induced substantial shifts in microbial community composition. Relative change analysis of dominant genera revealed that most taxa decreased in abundance following drought treatment. However, *Gaiella* and *Sphingomonas* exhibited positive relative changes, particularly in the drought-tolerant genotypes K111 and TGR78 (Figure 5A). In contrast, the sensitive genotype Bhutan showed more pronounced declines across multiple genera.

Venn diagram analysis demonstrated genotype and treatment specific patterns of shared and unique microbial species. Under drought stress, K111, TGR78, and Bhutan harbored 1,960, 1,499, and 1,469 unique species, respectively, with 1,406 species shared among all three genotypes. Under control conditions, the corresponding numbers of unique species were 1,160, 9,859, and 1,309, with 1,675 shared species (Supplementary Figures S2C,D). Differential abundance analysis revealed marked shifts in genus-level composition following drought treatment. The dominant genera *Pseudolabrys* and *Rhodoplanes* decreased across all three genotypes, with the largest reductions observed in Bhutan. In contrast, K111 and TGR78 exhibited smaller declines or partial retention of these taxa. Several genera, including *Novosphingobium*, *Marmoricola*, and *Mycobacterium*, increased specifically in TGR78 under drought conditions (Figure 5B).

Linear discriminant analysis effect size (LEfSe) further identified genotype-dependent differences in rhizobacterial composition. Under control conditions, *Sphingomonas and Pseudolabrys* were enriched in K111 and Bhutan, while *Gaiella* was enriched in Buhtan (Figure 5C). Under drought stress, *Anaeromyxobacter* was enriched in Bhutan, whereas *Nostoc* decreased in K111. Additionally, TGR78 showed enrichment of *Pseudolabrys* and *Rhodoplanes* under drought conditions (Figure 5D).

### 3.3 Differential metabolic responses of rice genotypes under drought stress

**Figure 6:**
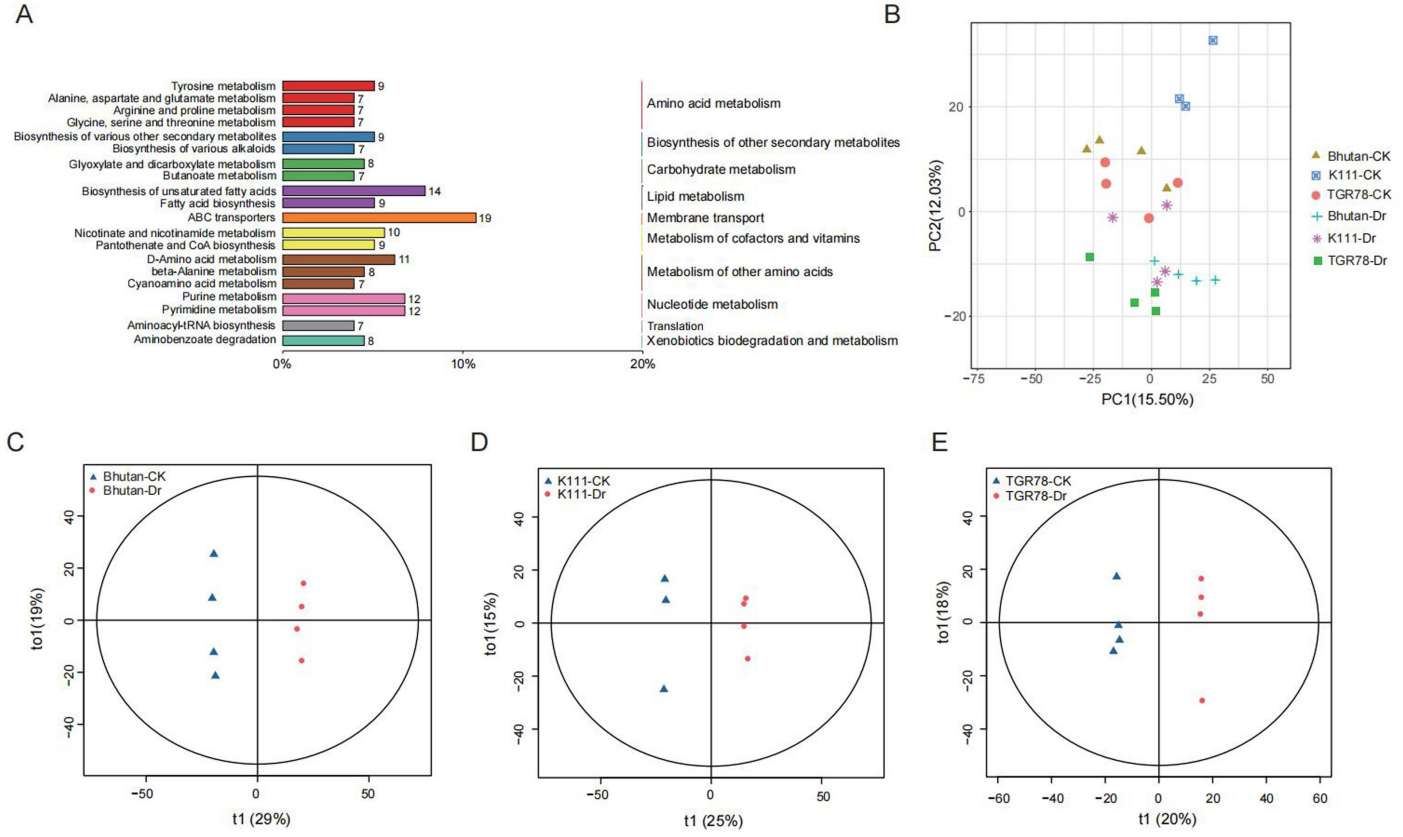
Multivariate and pathway analyses of rice metabolomic profiles under drought stress. **(A)** KEGG pathway enrichment analysis of differential metabolites **(B)** Principal component analysis (PCA) score plot showing separation of Bhutan-CK, K111-CK, TGR78-CK, Bhutan-Dr, K111-Dr, and TGR78-Dr, indicating distinct metabolic reprogramming among genotypes. **(C–E)** Partial least squares-discriminant analysis (PLS-DA) score plots for pairwise comparisons of Bhutan-CK vs. Bhutan-Dr, K111-CK vs. K111-Dr, and TGR78-CK vs. TGR78-Dr. Clear clustering patterns demonstrate metabolic divergence between drought-treated and control samples across all genotypes.

**Figure 7:**
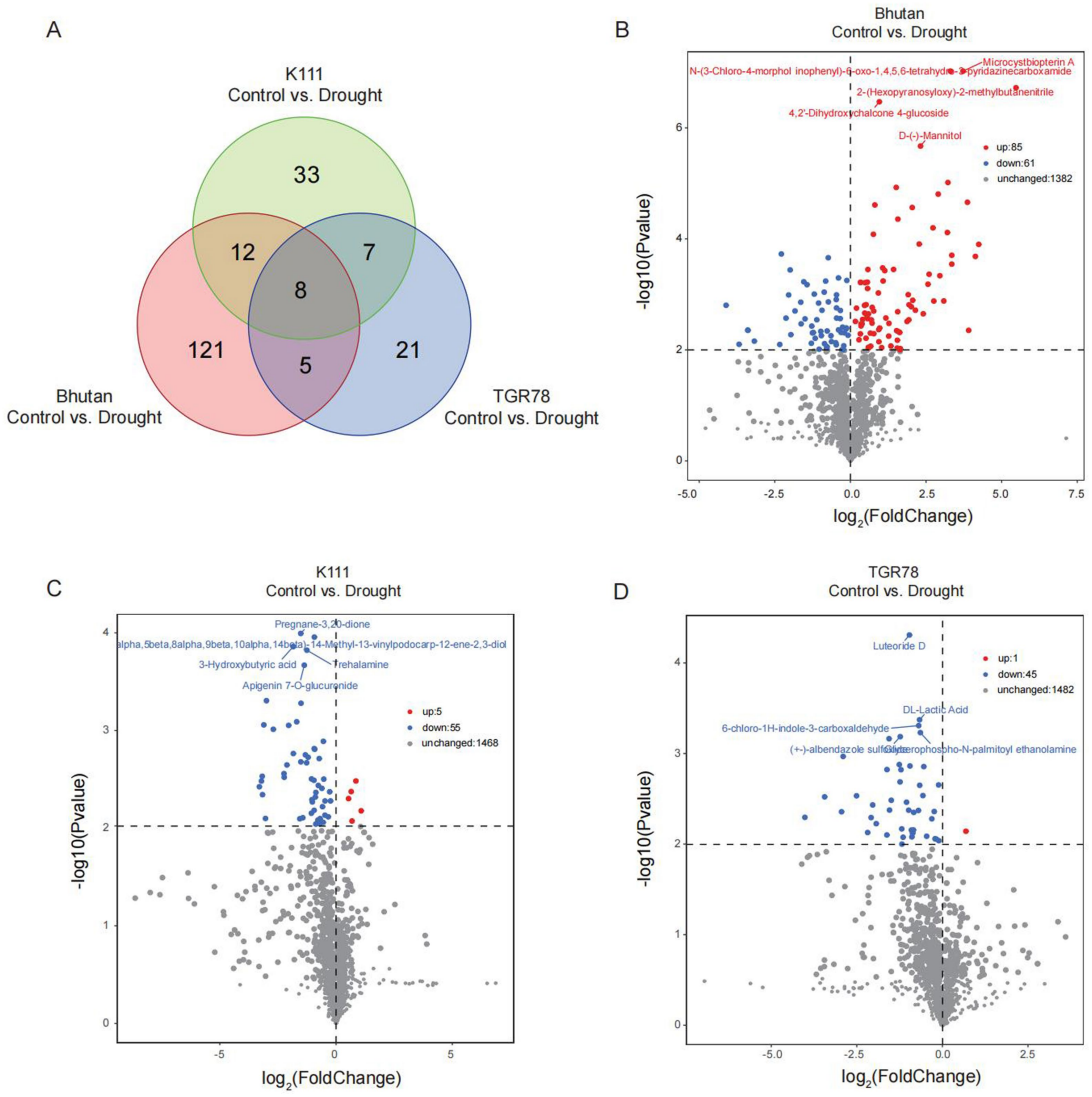
Differential metabolite analysis of three rice genotypes under drought stress. **(A)** Venn diagram showing the overlap of differential metabolites between Bhutan, K111, and TGR78 under control vs. drought conditions. **(B–D)** Volcano plots displaying upregulated (red), downregulated (blue), and unchanged (grey) metabolites in Bhutan, K111, and TGR78, respectively. Significantly changed metabolites were identified based on log, (fold change) and -log_10_ (*p* value) thresholds.

Untargeted metabolomic analysis using an Orbitrap LC–MS platform detected 1,674 peaks, of which 1,528 metabolites were successfully annotated. KEGG pathway enrichment analysis revealed that these metabolites were predominantly enriched in amino acid metabolism pathways, including tyrosine, alanine, aspartate, glutamate, arginine, proline, glycine, serine, and threonine. Pathways related to lipid metabolism (unsaturated fatty acid metabolism and fatty acid biosynthesis) and ABC transporters were also significantly enriched (Figure 6A). Principal component analysis of metabolite profiles revealed clear separation among the six sample groups, with PC1 and PC2 explaining 15.50 and 12.03% of the total variance, respectively. Drought-treated samples were distinctly separated from their corresponding controls (Figure 6B). Orthogonal partial least squares discriminant analysis (OPLS-DA) further confirmed these findings, demonstrating clear separation between control and drought treatments for all three genotypes (Figures 6C–E), indicating that drought stress induced substantial metabolic reprogramming in the rhizosphere. Venn diagram analysis showed genotype-specific patterns of metabolite sharing: 13 metabolites were shared between Bhutan and TGR78, 20 between Bhutan and K111, 15 between TGR78 and K111, and 8 among all three genotypes. Volcano plot analysis combined with fold change and variable importance in projection (VIP) screening identified 46 differential metabolites in TGR78, 60 in K111, and 146 in Bhutan (Figures 7A–D). These metabolites were primarily associated with amino acid metabolism, organic acid metabolism, and secondary metabolism, suggesting that these pathways were most responsive to drought stress.

The KEGG enrichment analysis and differential metabolite comparison between control and drought conditions revealed distinct metabolic responses among the three rice genotypes (Bhutan, K111, and TGR78). Bhutan exhibited extensive metabolic reprogramming, particularly in nucleotide metabolism, lipid metabolism, and energy-related pathways, with several metabolites such as Presecomycin B and 3-Hydroxyvaleric acid significantly upregulated under drought stress. K111 showed more moderate changes, with upregulation of metabolites like Pirin-3, primarily affecting purine metabolism and unsaturated fatty acid biosynthesis. TGR78 displayed the most severe metabolic perturbations, especially in nucleotide and secondary metabolism, with metabolites like (−) − 5′−desmethylyatein downregulated, suggesting a more focused metabolic response to drought stress. Overall, Bhutan underwent the most extensive and severe perturbations, highlighting its broader metabolic adjustment, while TGR78 showed more concentrated changes aimed at enhancing cellular stability under drought conditions.

### 3.4 Host–microbe–metabolite interactions under drought stress

**Figure 8:**
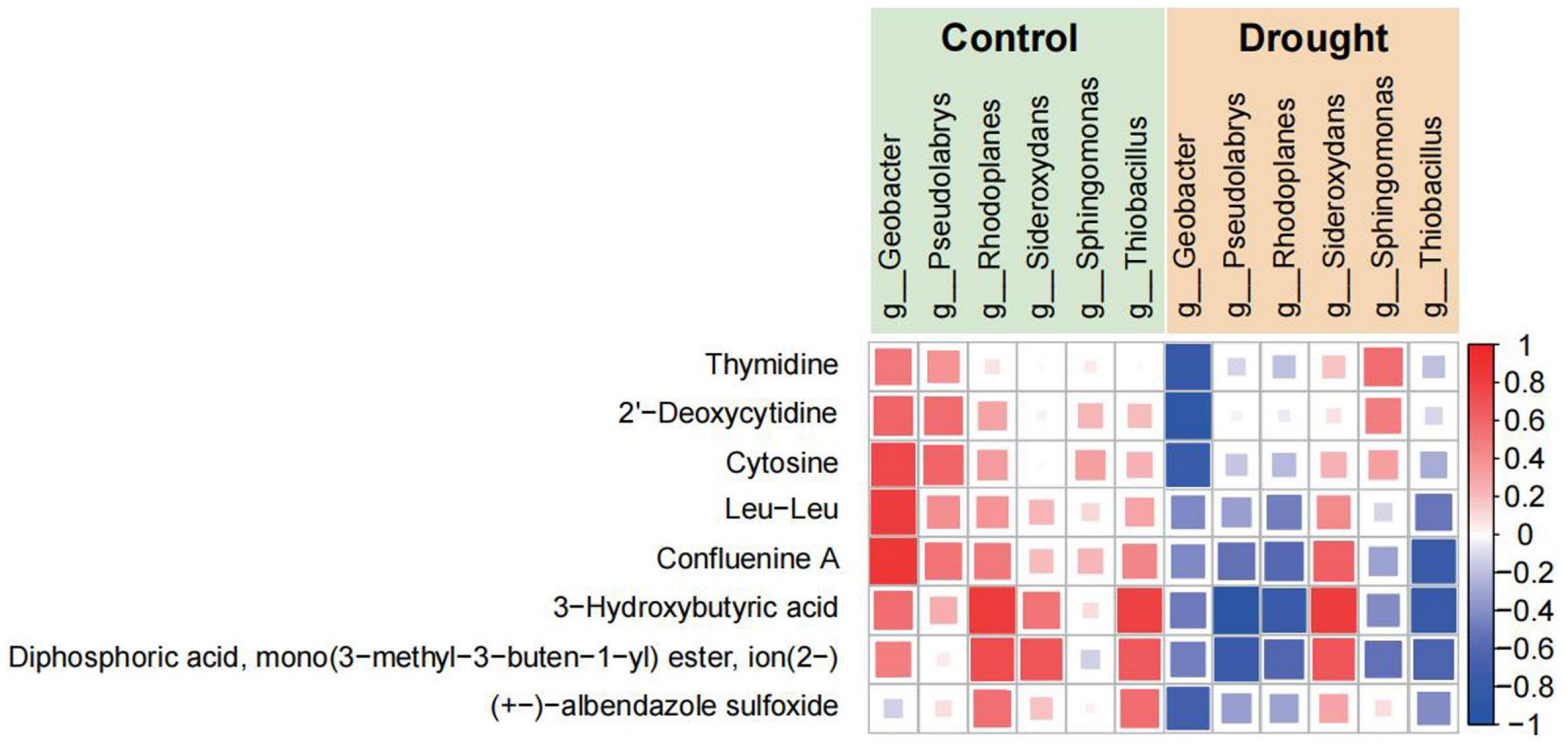
Correlation between microbial genera and metabolites under control and drought conditions. The heatmap shows the strength of the correlation between different microbial taxa (*Geobacter, Pseudolabrys, Rhodoplanes, Sideroxydans, Sphingomonas*, and *Thiobacillus*) and metabolites (e.g., thymidine, 2′-deoxycytidine, cytosine, Leu-Leu, confluenine A, 3-hydroxybutyric acid) in the rhizosphere. Red squares represent strong positive correlations, while blue squares indicate negative correlations. The data are presented for both control (green) and drought (orange) conditions. Significant differences in the microbe-metabolite interactions under drought stress highlight genotype-specific shifts in microbial community functions.

Correlation heatmap analysis revealed a significant reorganization of the associations between rhizosphere microbial genera and metabolites that coexisted in both the control and drought-induced modes. Under control conditions, most genera exhibited predominantly positive correlations with multiple metabolites. Under drought stress, this correlation structure shifted markedly, with several genera showing weakened positive associations or enhanced negative correlations, indicating a clear reorganization of the rhizosphere microbe-metabolite interaction network under water-deficit conditions. Among the drought-associated patterns, *Geobacter* showed a negative correlation with 3-hydroxybutyric acid. This relationship was consistent with the observed increase in *Geobacter* abundance under drought. For example, in TGR78, its relative abundance increased from approximately 3.5 to 4.8%, accompanied by a marked decrease in 3-hydroxybutyric acid, such as in Bhutan, where its abundance declined from approximately 1.45 × 10^8^ to 3.5 × 10^7^. In contrast, *Sphingomonas* exhibited a positive correlation with confluenine A, with both decreasing under drought conditions in the corresponding samples. Additionally, increased relative abundance of *Pseudolabrys* in K111 (from approximately 13 to 22%) and *Rhodoplanes* in TGR78 (from approximately 6 to 11%) was associated with decreased levels of several nucleotide-related metabolites (Figure 8; Supplementary Tables S2–S4). Collectively, these results demonstrate that drought stress altered rhizosphere microbe-metabolite associations and promoted more selective interaction patterns between microbial taxa and metabolites.

## 4 Discussion

Our study demonstrates that drought stress substantially restructures the rhizosphere ecosystem, with plant genotype playing a central role in shaping host-microbe-metabolite interactions. The transition from a highly connected microbe-metabolite network under well-watered conditions to a simpler and more drought-responsive network under stress is consistent with the view that environmental stress acts as an ecological filter, reducing interaction complexity while favoring a smaller set of functionally relevant associations (Toju et al., 2018; Trivedi et al., 2020). Importantly, this reduction in network complexity should not necessarily be interpreted as ecological deterioration. Rather, in the more resilient genotypes TGR78 and K111, it may reflect adaptive reorganization of the rhizosphere system in response to water deficit. The persistence of potentially beneficial taxa such as *Sphingomonas*, together with their positive associations with specific metabolites, suggests that maintenance of a coordinated microbial consortium may contribute to drought resilience. This interpretation is consistent with recent studies indicating that drought tolerance depends not only on microbial abundance, but also on the preservation of specific cooperative interactions that support host adjustment under stress (Wagner et al., 2016; Zhang et al., 2019; Ghosh et al., 2025).

One of the most important findings of this study is that drought-induced rhizosphere responses were strongly genotype dependent. Although all three rice genotypes showed shifts in microbial composition and metabolite profiles under drought stress, the magnitude and pattern of these changes differed substantially. In the drought-tolerant genotype TGR78, the rhizosphere community appeared comparatively more stable, and several taxa, including *Geobacter* and *Sphingomonas*, remained closely associated with specific metabolites under drought conditions. In particular, the enrichment of *Geobacter* together with the decline in 3-hydroxybutyric acid suggests a potential link between microbial enrichment and drought-associated metabolic reorganization. Although the underlying mechanism remains unclear, this pattern points to the possibility that specific microbial taxa contribute to the adjustment of rhizosphere carbon-related processes under water deficit. By contrast, the drought-sensitive genotype Bhutan exhibited broader metabolic perturbation and more widespread negative associations between microbial genera and metabolites, suggesting a less coordinated rhizosphere response to drought stress. These genotype dependent differences extend current understanding of plant-microbe interactions by indicating that host genetic background strongly influences the assembly and maintenance of stress-responsive rhizosphere communities (Fitzpatrick et al., 2018; Ullah et al., 2019).

The metabolic data further support the view that drought adaptation involves coordinated reprogramming of the rhizosphere environment. Compared with Bhutan, both TGR78 and K111 showed fewer differential metabolites and relatively more constrained metabolic shifts, suggesting greater stability of rhizosphere metabolic responses under drought. In contrast, the large number of differential metabolites detected in Bhutan indicates a broader disturbance of rhizosphere metabolic homeostasis. These differences imply that drought-tolerant genotypes may maintain a more regulated rhizosphere state, whereas drought-sensitive genotypes undergo more extensive metabolic disruption (Pérez-Jaramillo et al, 2018). When considered together with the microbial data, these findings support the idea that drought adaptation is associated with coordinated shifts in both rhizosphere community composition and metabolite accumulation, rather than changes in either component alone (Santos-Medellín et al., 2017).

Despite these advances, several limitations of the present study should be acknowledged. First, the relationships identified between microbial taxa and metabolites were based on correlation analyses and therefore do not establish causality. Functional validation will be required to determine whether the observed microbial taxa directly influence metabolite turnover or plant drought responses. Such validation could include gnotobiotic experiments, targeted metabolite supplementation, or inoculation with candidate strains. Second, our analysis was conducted at a single sampling time point, which limits our ability to resolve the temporal dynamics of rhizosphere community assembly and metabolite turnover during drought progression and recovery (Dai et al., 2019). Future studies should therefore combine longitudinal sampling with synthetic microbial community approaches to test the mechanistic contributions of key taxa such as *Geobacter* and *Sphingomonas* to drought-associated rhizosphere remodeling (Berendsen et al., 2018; Trivedi et al., 2020; Li et al., 2024). In addition, although this study focused on bacterial community responses and rhizosphere metabolites, other rhizosphere components, including fungi, protists, and non-biological soil chemical factors, may also contribute to drought-associated rhizosphere processes and should be considered in future work.

In conclusion, our integrated metagenomic and metabolomic analyses indicate that rice drought adaptation is associated with genotype-dependent restructuring of the rhizosphere host–microbe–metabolite network. A deeper understanding of these coordinated interactions will support the integration of rhizosphere-associated traits into future breeding and microbiome-assisted strategies for improving drought resilience in rice and other crop species.

## 5 Conclusion

Our findings reveal that drought stress does not merely degrade the rice rhizosphere but drives a genotype-specific reorganization of the host-microbe-metabolite network. We observed that while drought simplifies the complex connectivity seen under well-watered conditions, this represents a selective filtering process rather than ecological collapse, at least in resilient genotypes. TGR78 and K111 illustrate distinct survival strategies: TGR78 anchors itself through stable, synergistic associations with beneficial taxa like *Bradyrhizobium* and *Sphingomonas*, whereas K111 appears to leverage a reconfigured network to support elevated basal metabolism. Conversely, the drought-sensitive Bhutan genotype suffers from metabolic dysregulation and widespread negative microbial interactions, highlighting that tolerance is defined by the maintenance of cooperative networks rather than sheer microbial abundance. Although these correlative insights require further validation through longitudinal and gnotobiotic frameworks to establish causality, they fundamentally shift our focus toward breeding crops that can actively recruit and maintain protective rhizosphere communities under stress.

## Data Availability

Metagenomic datasets were deposited in the NCBI Sequence Read Archive (SRA) under accession number PRJNA1454352.
