# Supplementary figures and images for: Microbial and metabolic crosstalk in the rhizosphere shapes the divergent drought resilience of contrasting rice genotypes
Source: Front Microbiol. 2026 Apr 29;17:1788826. doi: 10.3389/fmicb.2026.1788826 (PMC13168078; doi:10.3389/fmicb.2026.1788826)

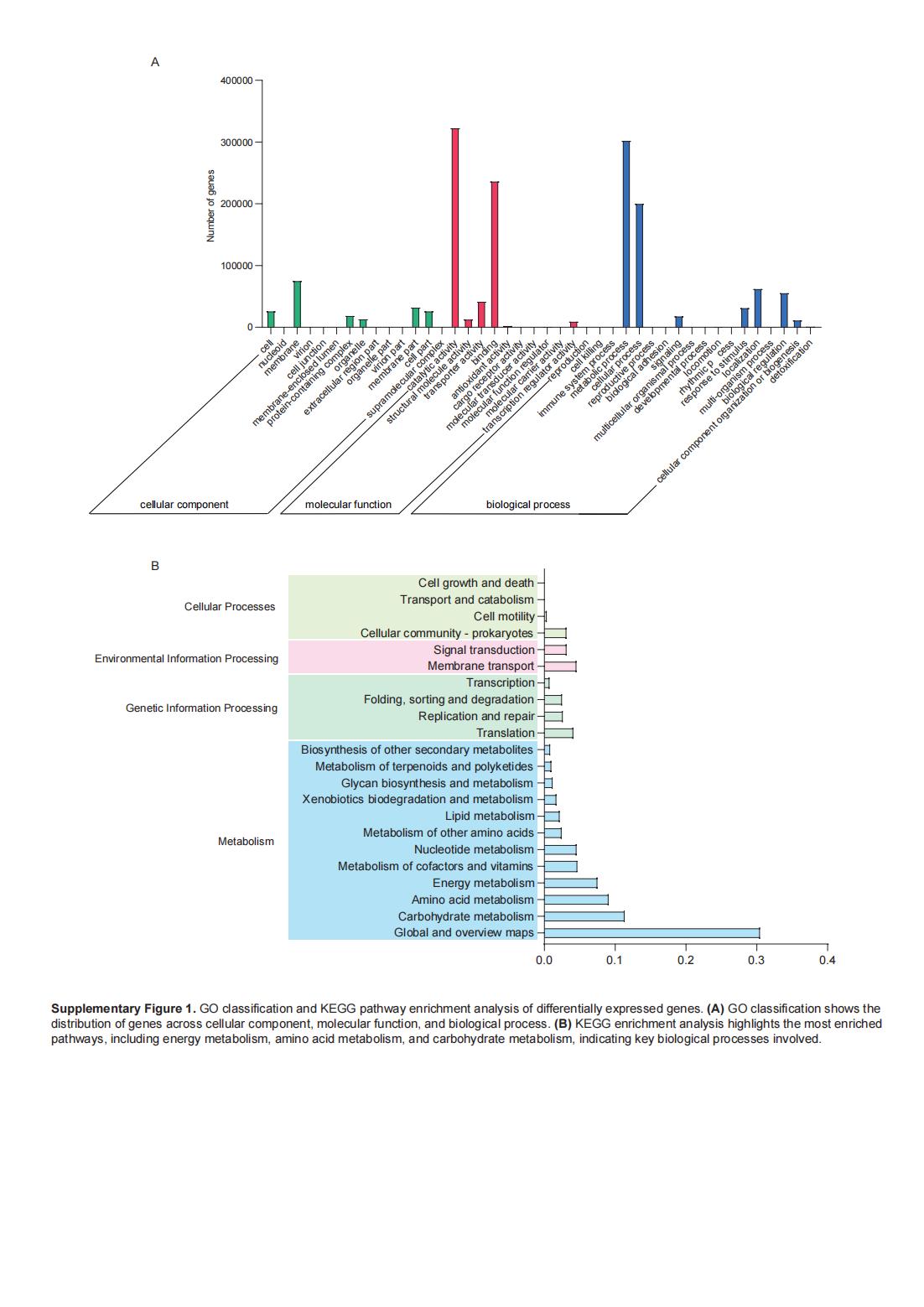

Supplement: Supplementary file 1 [file Image_1.JPEG]

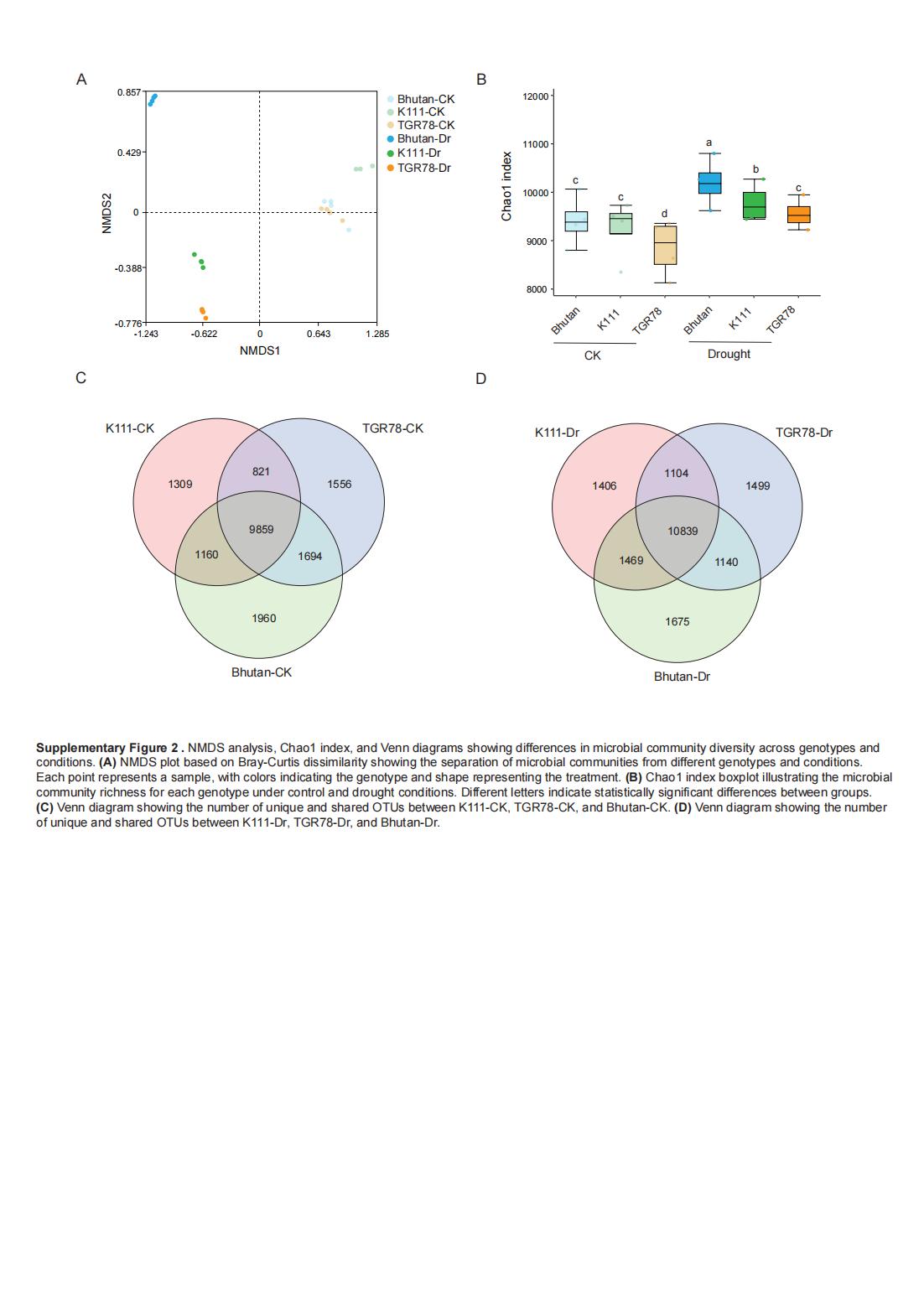

Supplement: Supplementary file 2 [file Image_2.JPEG]

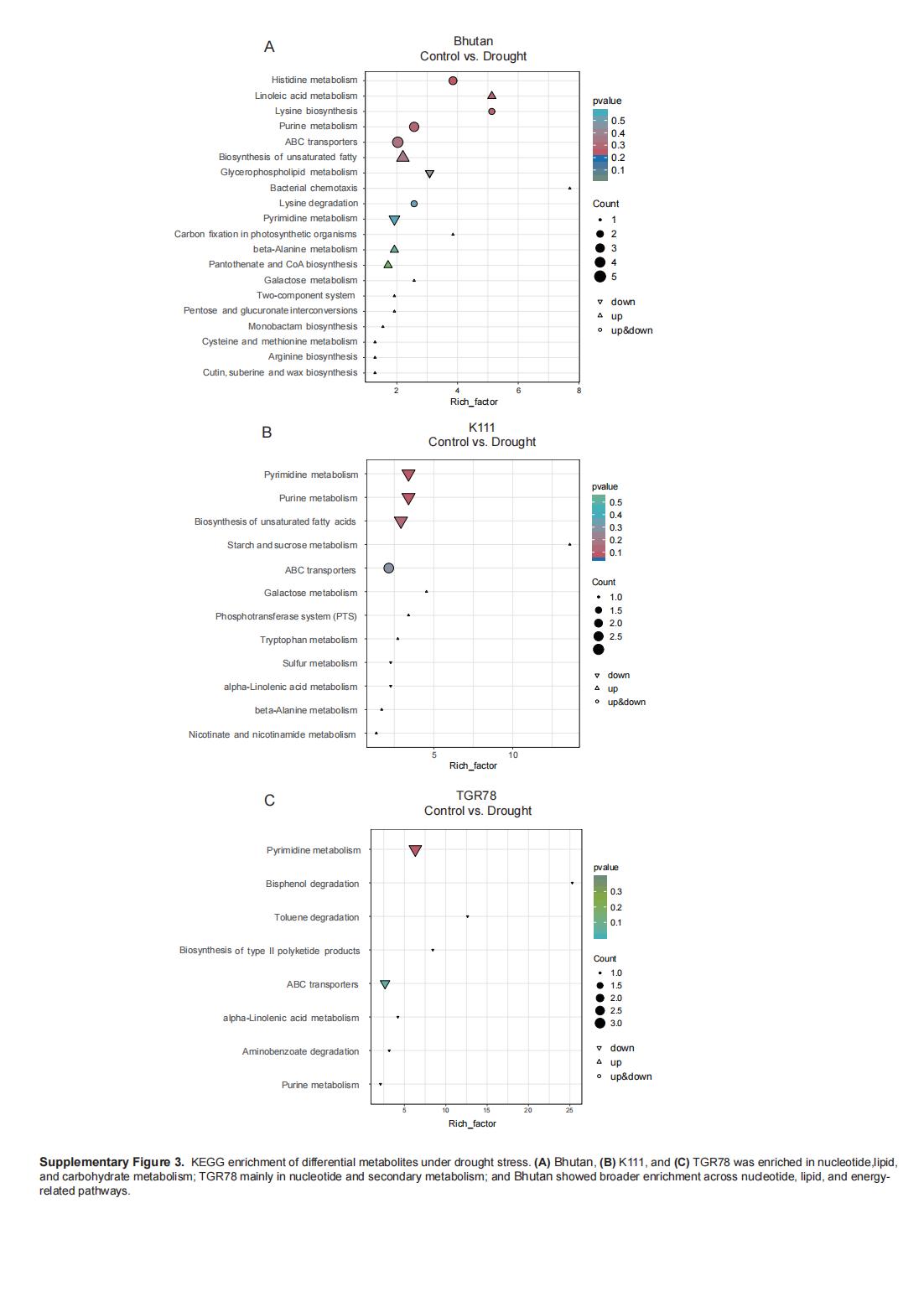

Supplement: Supplementary file 3 [file Image_3.JPEG]

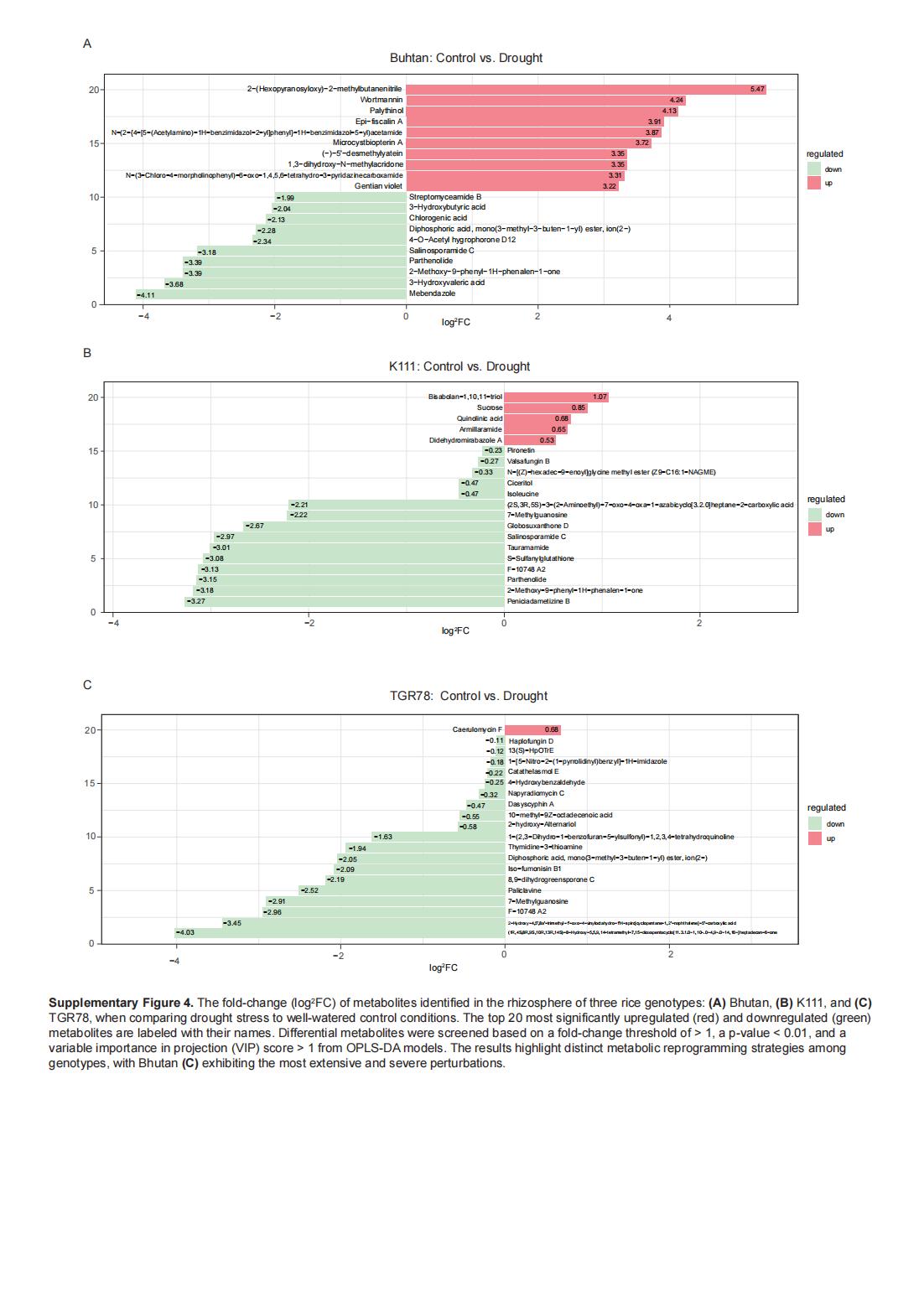

Supplement: Supplementary file 4 [file Image_4.JPEG]
