# Supplementary material for: Microbial and metabolic crosstalk in the rhizosphere shapes the divergent drought resilience of contrasting rice genotypes
Source: Front Microbiol. 2026 Apr 29;17:1788826. doi: 10.3389/fmicb.2026.1788826 (PMC13168078; doi:10.3389/fmicb.2026.1788826)
Supplement: Supplementary file 5 [file Table_1.DOCX]

Table 1. Metagenomic sequencing quality and output statistics for all experimental groups.

| Group | Mean Clean Reads (× 10⁸ reads) | Standard Deviation (× 10⁸ reads) | Range of Clean Reads (× 10⁸ reads) | Clean Data (Gb) | Standard Deviation (Gb) | Range of Data (Gb) | GC Content (%) | Q20 (%) | Q30 (%) |
| --- | --- | --- | --- | --- | --- | --- | --- | --- | --- |
| Buhtan-CK | 6.09 | ±0.23 | 5.82 ~ 6.36 | 40.68 | ±1.72 | 38.88~ 42.59 | 58.8 | 97.8 | 93.5 |
| Buhtan-Dr | 6.59 | ±0.23 | 6.26 ~ 6.83 | 43.71 | ±1.89 | 41.85~ 45.60 | 59.7 | 97.7 | 93.2 |
| K111-CK | 6.33 | ±0.17 | 6.40 ~ 6.81 | 44.21 | ±1.54 | 42.77~ 45.45 | 58.9 | 97.8 | 93.9 |
| K111-Dr | 6.69 | ±0.33 | 6.42 ~ 7.17 | 43.02 | ±1.67 | 41.04~ 44.83 | 59.5 | 97.6 | 93.6 |
| TGR78-CK | 6.16 | ±0.29 | 5.87 ~ 6.55 | 39.45 | ±1.23 | 38.19~ 40.39 | 58.9 | 97.7 | 93.7 |
| TGR78-Dr | 6.02 | ±0.13 | 5.86 ~ 6.16 | 39.98 | ±1.05 | 39.18~ 41.17 | 59.6 | 97.7 | 93.4 |

**Table 2.** Relative abundance of key genera across rice genotypes under control.

| sample | Bhutan-CK | K111-CK | TGR78-CK |
| --- | --- | --- | --- |
| *g__Anaeromyxobacter* | 0.0922 | 0.0871 | 0.0637 |
| *g__Bradyrhizobium* | 0.0937 | 0.0879 | 0.0741 |
| *g__Candidatus_Koribacter* | 0.0212 | 0.0202 | 0.0161 |
| *g__Candidatus_Sulfobium* | 0.1554 | 0.1665 | 0.1628 |
| *g__Candidatus_Sulfotelmatobacter* | 0.0230 | 0.0238 | 0.0205 |
| *g__Gaiella* | 0.1169 | 0.0834 | 0.0670 |
| *g__Geobacter* | 0.0341 | 0.0422 | 0.0350 |
| *g__Haliangium* | 0.0217 | 0.0218 | 0.0184 |
| *g__Ktedonobacter* | 0.0379 | 0.0172 | 0.0125 |
| *g__Marmoricola* | 0.0457 | 0.0097 | 0.0527 |
| *g__Novosphingobium* | 0.0043 | 0.0066 | 0.0707 |
| *g__Phenylobacterium* | 0.0174 | 0.0192 | 0.0488 |
| *g__Pseudolabrys* | 0.0749 | 0.1288 | 0.1128 |
| *g__Rhodoplanes* | 0.0518 | 0.0582 | 0.0422 |
| *g__Sideroxydans* | 0.0421 | 0.0164 | 0.0076 |
| *g__Sphingomonas* | 0.0871 | 0.1497 | 0.1450 |
| *g__Streptomyces* | 0.0436 | 0.0226 | 0.0287 |
| *g__Thiobacillus* | 0.0369 | 0.0386 | 0.0212 |

**Table 3.**Relative abundance of key genera across rice genotypes under drought.

| sample | Bhutan-Dr | K111-Dr | TGR78-Dr |
| --- | --- | --- | --- |
| *g__Sideroxydans* | 0.0255 | 0.0298 | 0.0134 |
| *g__Nostoc* | 0.0096 | 0.1305 | 0.0031 |
| *g__Sphingomonas* | 0.0500 | 0.0572 | 0.0604 |
| *g__Desulfobacca* | 0.0123 | 0.0110 | 0.0167 |
| *g__Pseudorhodoplanes* | 0.0132 | 0.0113 | 0.0134 |
| *g__Rhodoplanes* | 0.1073 | 0.0840 | 0.1139 |
| *g__Thiobacillus* | 0.0246 | 0.0234 | 0.0336 |
| *g__Candidatus_Sulfobium* | 0.1432 | 0.1278 | 0.1620 |
| *g__Pseudolabrys* | 0.2388 | 0.2147 | 0.2654 |
| *g__Geobacter* | 0.0533 | 0.0339 | 0.0473 |
| *g__Bradyrhizobium* | 0.1237 | 0.1051 | 0.1200 |
| *g__Anaeromyxobacter* | 0.1423 | 0.1208 | 0.0947 |

**Table 4.** Quantitative profiles of selected rhizosphere metabolites under control and drought stress.

| Name | Bhutan-  CK | Bhutan-  Dr | K111-CK | K111-Dr | TGR78-  CK | TGR78-  Dr |
| --- | --- | --- | --- | --- | --- | --- |
| Thymidine | 12533988 | 4016017 | 15111298 | 8951851 | 13387647 | 5646433 |
| 2'-Deoxycytidine | 7353670 | 2597921 | 9138300 | 4763629 | 7446731 | 3582899 |
| Cytosine | 27228782 | 11220768 | 35073386 | 18408086 | 27762651 | 13946958 |
| Leu-Leu | 2541935 | 1473428 | 3322826 | 1803597 | 2472839 | 1325413 |
| Confluenine A | 2049678 | 1131530 | 3065374 | 1479062 | 1962560 | 857113 |
| 3-Hydroxybutyric acid | 145338072 | 35286694 | 173548729 | 49043726 | 88827562 | 29974597 |
| Diphosphoric acid, mono(3-methyl-3-buten-1-yl) ester, ion(2-) | 5746372 | 1180005 | 6532773 | 1530298 | 3433290 | 831359 |
| (+-)-albendazole sulfoxide | 729133 | 250368 | 759697 | 377173 | 619292 | 262224 |
